# Knowledge and factors influencing schistosomiasis control interventions in the hyperendemic health district of Kalabancoro in Mali, 2020

**DOI:** 10.11604/pamj.2022.43.48.30512

**Published:** 2022-09-29

**Authors:** Fatoumata Koundou Maïga, Moussa Sangare, Housseini Dolo, Ilo Dicko, Abdoul Fatao Diabate, Modibo Keita, Lamine Diarra, Lamine Soumaoro, Sekou Thera, Oumar Diallo, Issa Guindo, Mahamadou Traoré, Ousmane Faye, Seydou Doumbia, Yaya Ibrahim Coulibaly

**Affiliations:** 1 Department of Education and Research in Public Health and Specialties, Faculty of Medicine and Odontostomatology of Bamako, Bamako, Mali,; 2 Kalabancoro Reference Health Center, Koulikoro Region, Koulikoro, Mali,; 3 Filarisis Unit, International Center of Excellence in Research, Bamako, Mali,; 4 Interdisciplinary School of Health Sciences, Faculty of Health Sciences, University of Ottawa, Ottawa, Canada,; 5 University Clinical Research Center, Point G, Bamako, Mali,; 6 Hellen Keller International Bureau du Mali, Bamako, Mali; 7 Population Service International Mali, Population Services International, Bamako, Mali,; 8 Programme National de Lutte contre les Schistosomiases et les Géo-Helminthiases, Bamako, Mali,; 9 Hôpital de Dermatologie de Bamako, Bamako, Mali

**Keywords:** Schistosomiasis, knowledge, Kalabancoro, children, adult, Mali

## Abstract

**Introduction:**

schistosomiasis is a public health concerns in many countries including Mali. In Kalabancoro District, during the 2017 assessments, the National schistosomiasis and soil-transmitted helminths control program reported prevalence´s of 10.83% and 50.83% for urinary schistosomiasis and intestinal schistosomiasis respectively. This district recorded the highest prevalence of intestinal schistosomiasis among the 46 districts evaluated. To better understand these high rates, this study investigated the knowledge of schistosomiasis in children and adults in this district.

**Methods:**

a cross-sectional study was conducted which involved 947 participants. A univariate analysis and multiple logistic regression were performed. Data collection was through questionnaire administration.

**Results:**

during the study, 76.1% of participants claimed to know about schistosomiasis (p<0.001) among them, 85.6% did not know the mode of contamination (p=0.001) and 66.3% knew the traditional treatment (p=0.004). Participants whose households were close to water impoundment were 2.16 times more likely to know schistosomiasis than those who were not (95% CI = [1.49 - 3.11]).

**Conclusion:**

most of the majority of participants reported being aware of schistosomiasis. However, the modes of transmission, prevention, and treatment of schistosomiasis were not well known. Misconceptions persist, hindering effective prevention and control. This is a tangible obstacle to the elimination of schistosomiasis in the Kalabancoro Health District and requires interventions tailored for these endemic communities.

## Introduction

Schistosomiasis is a parasitic disease caused by trematodes of the genus *Schistosoma* [1]. It is a disease of poverty, and unequally affects the less wealthy people of the world [2]. This water-borne disease is linked to poor hygiene conditions in the population, mainly in children aged 7 to 14 years [3]. Infestation can occur when humans come into contact with schistosomes in their freshwater larval form, cercariae, via infested water sources [3]. Schistosomiasis remains a major public health problem in many countries of sub-Saharan Africa [4,5]. It represents the second most devastating parasitic disease after malaria [6]. Still endemic in 74 countries, the infection affects 261 million people worldwide with nearly 800 million people exposed [6,7]. More than 90% of these cases live in Sub-Saharan Africa. The United Republic of Tanzania carries the second highest burden after Nigeria, with prevalence estimates ranging from 12.7% to 87.6% [1].

In Mali, the overall prevalence was 30% in 2019 [6]. The hyperendemic areas are the irrigated lands of the Office du Niger in Segou, the Plateau Dogon with small dams and the Senegal River basin which had a prevalence of over 50% in 2006 [6]. In 2005, preventive chemotherapy through mass drug administration (MDA) campaigns with praziquantel (PZQ) (40 mg/kg) was effectively implemented in Mali [3]. Despite these annual or biannual treatments with the required coverage rates, it has been reported that reinfection occurs in treated children 12 months in endemic areas [8]. Many studies have reported that the high prevalence of schistosomiasis is associated with lack of knowledge of how to prevent the disease [9-11]. In 2018, a study conducted in Togo showed that 40.10% of the population did not know the mode of transmission of the disease. This proportion was also unaware that avoiding contact with contaminated surface water could prevent the disease [10]. In Mali, a study conducted in a peri-urban school in Bamako reported that the causes of terminal hematuria were unknown by 64.6% of the children surveyed [11]. In the Kalabancoro health district, during the most recent assessments in 2017, the National Schistosomiasis and soil-transmitted helminths Control Program (PNLSH) reported a prevalence of urinary schistosomiasis of 10.83% and a prevalence of intestinal schistosomiasis at 50.83%. This district had the highest rate of intestinal schistosomiasis among the 46 districts evaluated. These high prevalence´s in Kalabancoro could be due to lack of awareness of schistosomiasis prevention and also to activities related to surface water such as fishing, sand mining, and others daily activities in the river. To date, no study has been conducted on the knowledge of schistosomiasis in the Kalabancoro health district of Mali, hence this study reported on the factors associated with the lack of knowledge of schistosomiasis in the Kalabancoro health district.

## Methods

**Study design and period**: this was a cross-sectional study conducted from May to November 2020.

**Figure 1:**
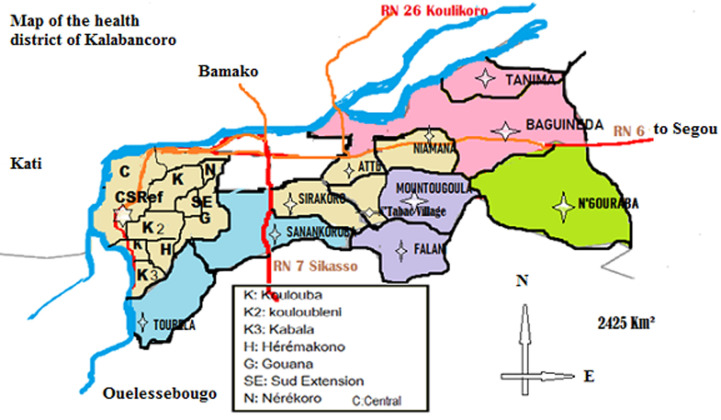
map of Kalaban-coro health district

**Study site**: this study was conducted in the health district Kalabancoro (Figure 1) located in the second administrative region of Mali (Koulikoro) close to Bamako the capital city. This district is located on the right bank of the Niger River and covers an area of 25,425 km^2^ with 117 villages and a total population of 349,970 inhabitants in 2020. In this district, the economy is based on agriculture, livestock, handicrafts and trade. In addition, fishing in the Niger River offers the population a wealth of fish, but also an opportunity to exploit the sand and gravel used in building houses and roads [12].

**Population and participant selection procedure**: the study involved children aged of 9-14 years and a group of 15 years and older, which we referred to as adults for the purposes of this study. Two stage sampling (villages and households) was conducted. The first stage of sampling consisted of identifying the study villages. Each village had an equal probability of being selected. Thirty (30) clusters/villages were randomly selected using systematic random sampling. The second stage of sampling consisted of identifying the households to be surveyed in each village. The selected households were not replaced when residents were absent. To minimize the number of absent residents in the selected households, survey teams revisited the household before leaving the village on the day of the survey. Once households were identified, all eligible volunteers of the household were surveyed.

**Data collection tools and procedures**: a digitalized questionnaire was used to collect data from households through the open data kit (ODK) collect platform using smartphones [13]. Eight seventh-year medical school students from the Faculty of Medicine and Odonto-Stomatology of Bamako were trained for electronic data collection with smartphones. This data collection was done over a weeklong period. The study team collected socio-demographic information and then assessed the level of knowledge about schistosomiasis.

**Sample size**: the sample size was calculated according to the following formula with a precision of 5% [14].


$$N=D*\frac{Z^{2}(P*Q)}{i^{2}}$$

Where N = minimum sample size, P = prevalence of knowledge of schistosomiasis in Malian community = 0,354 Q =1-P = 0,646 i = precision = 0.05, Z = reduced variance = 1.96, D = cluster effect = 2, then N=2*((1.96)^2^*(0.354*0.646))/((0.05)^2^) = 703. The non-participation rate was estimated at 20% = 702*20/100 = 141. The minimum required sample size for the study was 844.

**Data analysis**: socio-demographic data such as age, sex, village of residence, level of education, type of school attended, main activity and marital status were collected. The knowledge of modes of contamination, modes of prevention, treatment, and knowledge and participation to mass drug distribution targeting neglected tropical diseases (NTDs) were also assessed. For data analysis, Statistical Package for Social Sciences (SPSS) version 25.0 was used. Quantitative variables were expressed in terms of median and minimum-maximum. Categorical variables were expressed in terms of numbers and percentages. Pearson´s Chi-square test or Fisher´s exact test with α risk of 5% was used for comparison of proportions if applicable. Multivariate analysis, binary logistic regression adjusted for co-variates were done using the Backward elimination selection method to study the binary dependent variation [15]. The results were presented in tabular form with the raw and adjusted Odds ratios with their 95% confidence intervals and p-values. The Hosmer-Lemeshow test was used to assess the consistency of the selected regression model [16].

**Ethical considerations**: the ethics committee of the Faculty of Medicine, Pharmacy and Odontostomatology approved the study under the number 2019/39/CE/FMPOS. In the field, permission was obtained from the administrative and traditional authorities, parents or guardians of the children, and the community was informed of all aspects of the survey. The consent of participant was obtained before the data collection. All information collected on the participants, personal data was confidential with limited access to the investigators.

## Results

**Socio-demographic characteristics**: a total of 947 participants were surveyed, of them 50.4% (477/947) were male and 49.6% (470/947) were female. The median age of the participants was 26 years with extremes of 9 and 89 years. The 15 years and older represented 73.6% (697/947) of the study population. Subjects aged 15-34 years were the most represented among study participants (38.1% (361/947)) while those aged 9-11 years were the least common (11.8% (112/947)). At the study site level, 17.2% (62/361) of 15-34-year-olds and 38.7% (130/336) of those over 34 years of age were farmers (p<0.001). Students accounted for 92% (103/112) among 9-11-year-olds and 91.3% (126/138) among 12-14-year-olds (p=0.29). A proportion of 65.5% (620/947) of participants were enrolled in school. However, the enrollment rate among children was 90.8% (227/250) while it was 56.4% (393/697) among adults (Table 1).

**Table 1:** sociodemographic characteristics

Variables	Adults		Children		Total	
	n	%	n	%	n	%
**Sex**						
Male	347	49.8	130	52	477	50.4
Female	350	50.2	120	48	470	49.6
**Total**	**697**	**100**	**250**	**100**	**947**	**100**
**Age group (year)**						
15-34	361	51.8	0	0	361	38.1
35 and plus	336	48.2	0	0	336	35.5
9-11	0	0	112	44.8	112	11.8
12-14	0	0	138	55.2	138	14.6
**Total**	**697**	**100**	**250**	**100**	**947**	**100**
**Main activities**						
Student	64	9.2	229	91.6	293	30.9
Farmer	192	27.6	1	0.4	193	20.4
Fisherman	10	1.4	0	0	10	1.1
Market gardener	47	6.7	1	0.4	48	5.1
Gold digger	2	0.3	0	0	2	0.2
Sand farmer	25	3.6	3	1.2	28	2.9
Others	357	51.2	16	6.4	373	39.4
**Total**	**697**	**100**	**250**	**100**	**947**	**100**
**Education**						
Yes	393	56.4	227	90.8	620	65.5
No	304	43.6	23	9.2	327	34.5
**Total**	**697**	**100**	**250**	**100**	**947**	**100**

n = Number, Other (main activities) = Farmer, Tailor, Shopkeeper, Housekeeper, Carpenter, Hairdresser, Teacher, Mason

**Figure 2:**
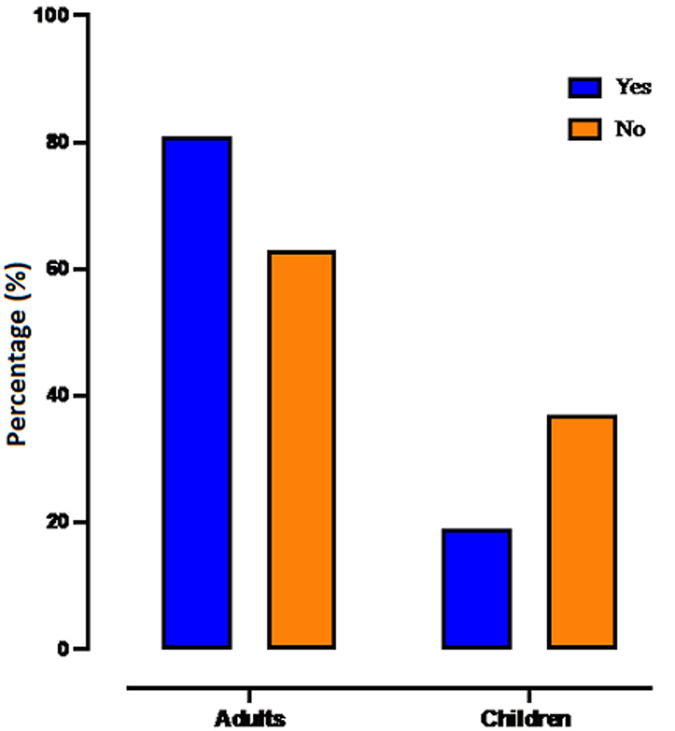
knowledge of schistosomiasis by the study population in Kalabancoro district in 2020

**Figure 3:**
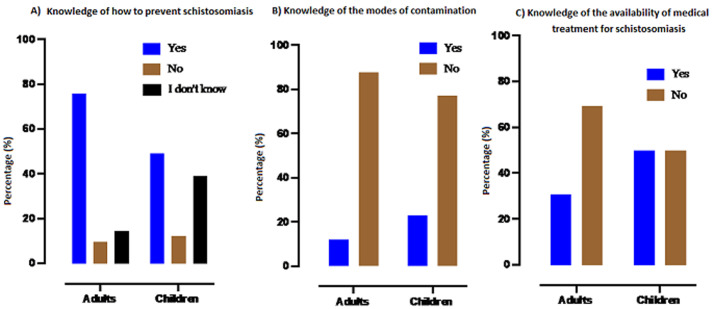
level of knowledge of schistosomiasis by the study population in Kalabancoro district in 2020

**Knowledge about schistosomiasis**: among the 15 years old and above, 80.9% (564/697) claimed to know schistosomiasis as did 62.8% (157/250) of children (p<0.001) (Figure 2). But, in the study population, 87.9% (496/564) of the 15 years old and above and 77.1% (121/150) of children did not know the mode of transmission of schistosomiasis (p<0.001). However, 75.9% of the 15 years old and above versus 49% of children thought the disease was preventable (p<0.001). Of the 374 participants (39.5% of the study population) who said they knew treatment for schistosomiasis, 66.3% (248/374) spoke about traditional treatment (p = 0.004) (Figure 3).

**Factors influencing schistosomiasis control interventions**: after adjusting for other confounding factors, we found age, gender, and household next to a water impoundment significantly associated with knowledge of schistosomiasis. Men were 3.47 times more likely to know about schistosomiasis than women [aOR=3.47 (95% CI= 2.48-4.85)]. Adults aged 15 years and older were 2.72 times more likely to experience schistosomiasis than children aged 9 to 14 years [aOR=2.72 (95% CI= 1.94-3.82)]. Participants whose households were next to a water impoundment were 2.15 times more likely to experience schistosomiasis than participants who were not next to a water impoundment [aOR=2.15 (95% CI= 1.49-3.11)] (Table 2). After adjustment for confounding factors, the variables existence of effective treatment, age, and confidence in MDA drugs were significantly associated with the habit of participation to MDA. Children were 4.08 times more likely to have participated in MDA than adults [aOR=4.08 (95% CI= 1.31-12.6)]. Respondents who trusted MDA drugs were 8.50 times more likely to have participated in MDA than those who did not trust the products [aOR=8.50 (95% CI= 4.72-15.30)]. Those who believed that an effective treatment existed were 1.77 times more likely to have participated in MDA than those who believed that an effective treatment did not exist [aOR=1.77 (95% CI= 1.03-3.04)] (Table 3).

**Table 2:** factors associated with knowledge of schistosomiasis among adults and children in Kalabancoro District in 2020

Variables	OR [IC 95%]	ORa [IC 95%]
**Age group**		
Children	(1)	(1)
Adults	2.51 [1.82-3.45]	2.72 [1.94-3.82]
**Sex**		
Female	(1)	(1)
Male	3.30 [2.39- 4.56]	3.47[2.48-4.85]
**Household next to a water reservoir**		
No	(1)	(1)
Yes	2.31[1.63-3.28]	2.15 [1.49-3.11]

(1)= reference, Hosmer-Lemeshow test [p = 0.279], ORa= adjusted odds ratios

**Table 3:** factors associated with praziquantel uptake among adults and children during mass treatment campaigns in Kalabancoro district in 2020

Variables	OR [IC 95%]	ORa [IC 95%]
**Evidence of terminal hematuria in the past**		
No	(1)	(1)
Yes	1.57 [0.93 -2.62]	1.691 [0.93 - 3.076]
**Existence of effective treatment**		
No	(1)	(1)
Yes	1.65 [1.03 - 2.66]	1.77 [1.03 - 3.04]
**Age group**		
Adults	(1)	(1)
Children	1.95 [1.11- 3.41]	4.081 [1.31 - 12.64]
**Confidence in NTDs/MDA products**		
No	(1)	(1)
Yes	5.23 [3.37-8.12]	8.50 [4.72 - 15.30]

## Discussion

This study was conducted in communities where schistosomiasis is hyper endemic and where, despite the implementation of prevention measures including mass treatment, infection persists. The results of this study will be discussed in terms of factors associated with knowledge of the disease, factors influencing participation in ongoing interventions including mass treatment. During this study, more than 75% of participants claimed to know about schistosomiasis, among them more than 85% did not know the mode of infection (p=0.001). However, more than 66% of those who claimed to know about schistosomiasis, knew the traditional treatment (p=0.004). Participants whose households were near a water reservoir were more than 2 times more likely to know about schistosomiasis than those who were far away (95% CI = [1.49-3.11]).

Our study showed that male participants were more than 3 times more likely to know about schistosomiasis than female participants (p<0.001). This would be due to the fact that the female has less access to information than the male perhaps because of the difference in formal education between men and women in Mali [17]. Participants of 15 years of age and above were about 3 times more likely to know about schistosomiasis than participants of 9 to 14 years of age (p <0.001). The knowledge of this population group would be explained by their maturity and also the shyness of some 9 to 14-year-olds to say “I don't know” even if they knew the answers to the questions. Participants whose households were next to a water impoundment were more than 2 times more likely to know about schistosomiasis than participants who were far from it (p <0.001). This could be because they are more likely to experience schistosomiasis (as these households would be more likely to frequent these reservoirs for household chores and daily needs, thus behaving in ways that put them at greater risk) [18]. It would be better for the NTD programs to focus their sensitization and awareness campaigns on women and the population closes to water impoundment during and after the MDA. We also suggest involving women association leaders in the organization of MDA campaigns.

Respondents who believed that an effective treatment exists were about 2 times more likely to participate in NTD/MDA than those who believed that an effective treatment does not exist (p = 0.03). We think that because most of them believe in modern medicine say that there is an effective treatment even if they don´t know. Children were more than 4 times more likely to participate in NTD/MDA than adults (p < 0.01), which could be explained by the fact that schools are prioritized during the campaigns. Pressure of attending from teachers and also the mass effect (seeing other peers taking the medication) also support this observation [19]. Respondents who were confident in MDA medicines were more than 8 times more likely to participate in MDA than those who were not confident (p <0.001). Which is quite normal because to participate in a campaign you need to be confident on the whole process including the medicines [20]. To take this in consideration, we suggest making a preliminary survey into each community to capture community knowledges attitudes and practices before implementing any activities. That may help to better communicate our messages. More than half of the participants were educated (65.5%). This rate was more than 90% for children and 56% for adults. This could be explained by an improvement in school enrollment rates over time as a result of mobilizing measures by the authorities during the last decade. Also, Kalabancoro the study area is one of the big cities of Mali and is very close to the capital Bamako. The majority (>76%) of respondents indicated that they knew about schistosomiasis, using the most common local term (*sougounébileni, massadimi, grossian or damadialan*). Most of the participants links the disease with gastric ulcers and sexually transmitted infections (STIs). Others believe that schistosomiasis is caused by exposure to the sun and donkey urine. Our result is in line with the findings of a study by Rassi *et al*. (2016) who found that 91% of their participants knew about schistosomiasis with local terms and have different explanations about the origin of the disease [20].

In this study, 70% of respondents who were aware of schistosomiasis reported knowing how to avoid the disease. Adults (>75%) were more informed than children (49%). This is due to the maturity of the participants aged 15 years and older compared to the participants aged 9 to 14 years. This result contrasts with that of Barrow *et al*. (2020) who reported that 45.3% of their respondents reported not knowing any method of prevention against schistosomiasis [18]. Less than a quarter (14.4%) of the participants in the present study knew the mode of transmission of the disease. This would be due to the fact that in the peri-urban community, many people believe that the disease is related to prepuberty and also to prolonged sun exposure. This result contrasts with that of Mwai *et al*. (2016) who found that only 14.04% of respondents did not know the means of transmission [21]. In the study population, those who knew that treatment was available, only about 33% mentioned medical treatment. This result could be explained by the poor peri-urban community's reliance on traditional treatments rather than medical treatment [19,22]. Our result differs from that of Christian Rassi *et al*. (in Mozambique in 2016) who said that among their respondents who knew about schistosomiasis, more than half (61%) reported that they did not know if there was a medicine that treats the disease and 8% had reported that such a medicine did not exist although 28% had reported that there was a medicine [20].

**Limitations and challenges**: the external validity of this study is reduced because several villages declined to participate in the survey because of the 2019 coronavirus pandemic. To replace these villages, other nearby villages were randomly selected to replace them. The short time frame of the study may have made it impossible to include some community members with activities or habits that require them to be out of the household during the time of our visit (June, beginning of the rainy season) or out of the household during the day.

## Conclusion

The majority of respondents claimed to be familiar with schistosomiasis. However, details about the cause, prevention and treatment of schistosomiasis were not well known. It was found that the community tended to rely on traditional remedies and that persistent misconceptions hindered effective prevention and control of the disease. This could be a real obstacle to schistosomiasis control in the Kalabancoro health district. Therefore, it´s important to sensitize the communities on the mode of transmission, prevention and treatment of schistosomiasis and to put in place interventions targeting children and women that can reduce the level of transmission.

### What is known about this topic

- In the various studies, many people still use surface water for their daily activities;
- Water activities are the main risk factors for reinfection of the population;
- Knowledge of schistosomiasis among the population is inadequate and insufficient.

### What this study adds

- This study provides information on Knowledge and factors influencing the interventions to control schistosomiasis in the hyperendemic health district of Kalabancoro in Mali, 2020;
- Most respondents claimed to know about schistosomiasis, however, details about the cause, prevention and treatment of schistosomiasis were not well known;
- Our study also identified factors associated with knowledge of schistosomiasis in the health district of Kalabancoro in Mali, 2020.
